# Supplementary material for: Model-based deep embedding for constrained clustering analysis of single cell RNA-seq data
Source: Nat Commun. 2021 Mar 25;12:1873. doi: 10.1038/s41467-021-22008-3 (PMC7994574; doi:10.1038/s41467-021-22008-3)
Supplement: Supplementary file 1 — Supplementary Information [file 41467_2021_22008_MOESM1_ESM.pdf]

# Supplementary Figures

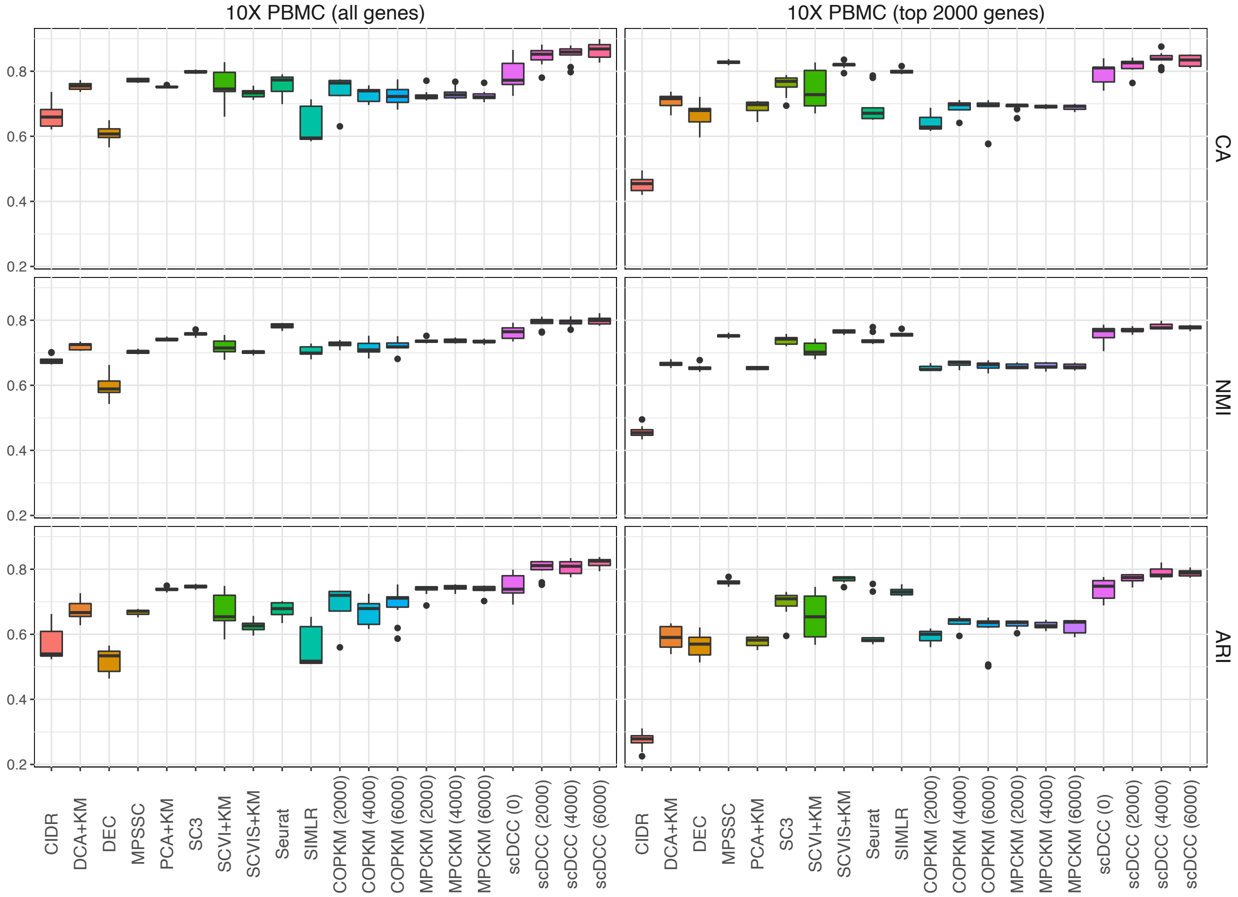


**Figure S1.** Clustering performances of different methods on the 10X PBMC dataset (randomly selected 2100 cells), measured by NMI, CA and ARI. The number in parentheses is the number of pairwise constraints. All experiments are repeated for ten times. The lower, middle, and upper hinges correspond to the first, second and third quartiles (the 25th, 50th and 75th percentiles). Let IQR be the distance between the first and third quartiles. The upper whisker extends from the hinge to the largest value no further than 1.5 * IQR from the hinge, while the lower whisker extends from the hinge to the smallest value at most 1.5 * IQR from the hinge. Data points beyond the end of the whiskers are outliers and plotted individually. Note that we use “KM” to represent “k-means” to save space.

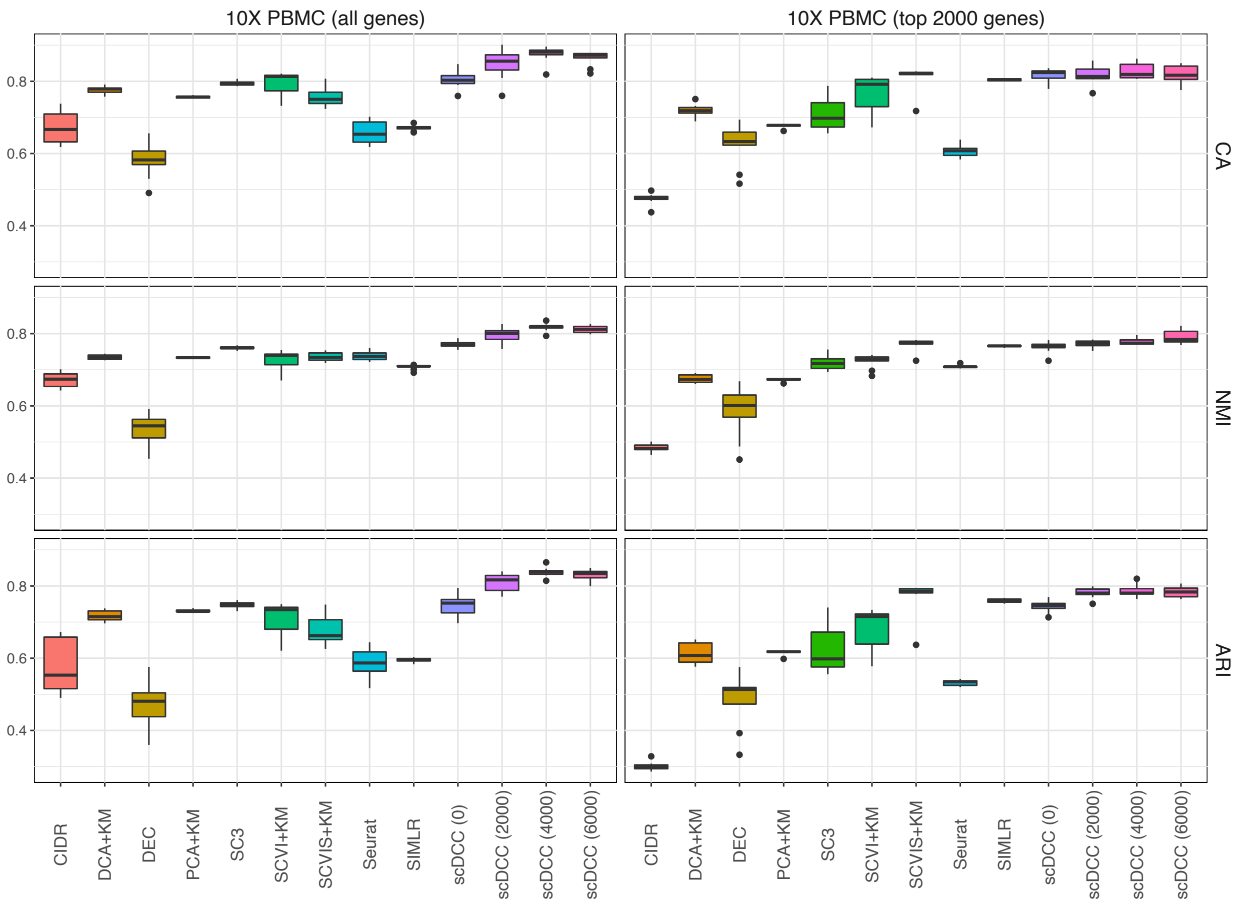


**Figure S2.** Clustering performances of different methods on the 10X PBMC dataset (full dataset), measured by NMI, CA and ARI. The number in parentheses is the number of pairwise constraints. All experiments are repeated for ten times. The lower, middle, and upper hinges correspond to the first, second and third quartiles (the 25th, 50th and 75th percentiles). Let IQR be the distance between the first and third quartiles. The upper whisker extends from the hinge to the largest value no further than 1.5 * IQR from the hinge, while the lower whisker extends from the hinge to the smallest value at most 1.5 * IQR from the hinge. Data points beyond the end of the whiskers are outliers and plotted individually. Note that we use "KM" to represent "k-means" to save space.

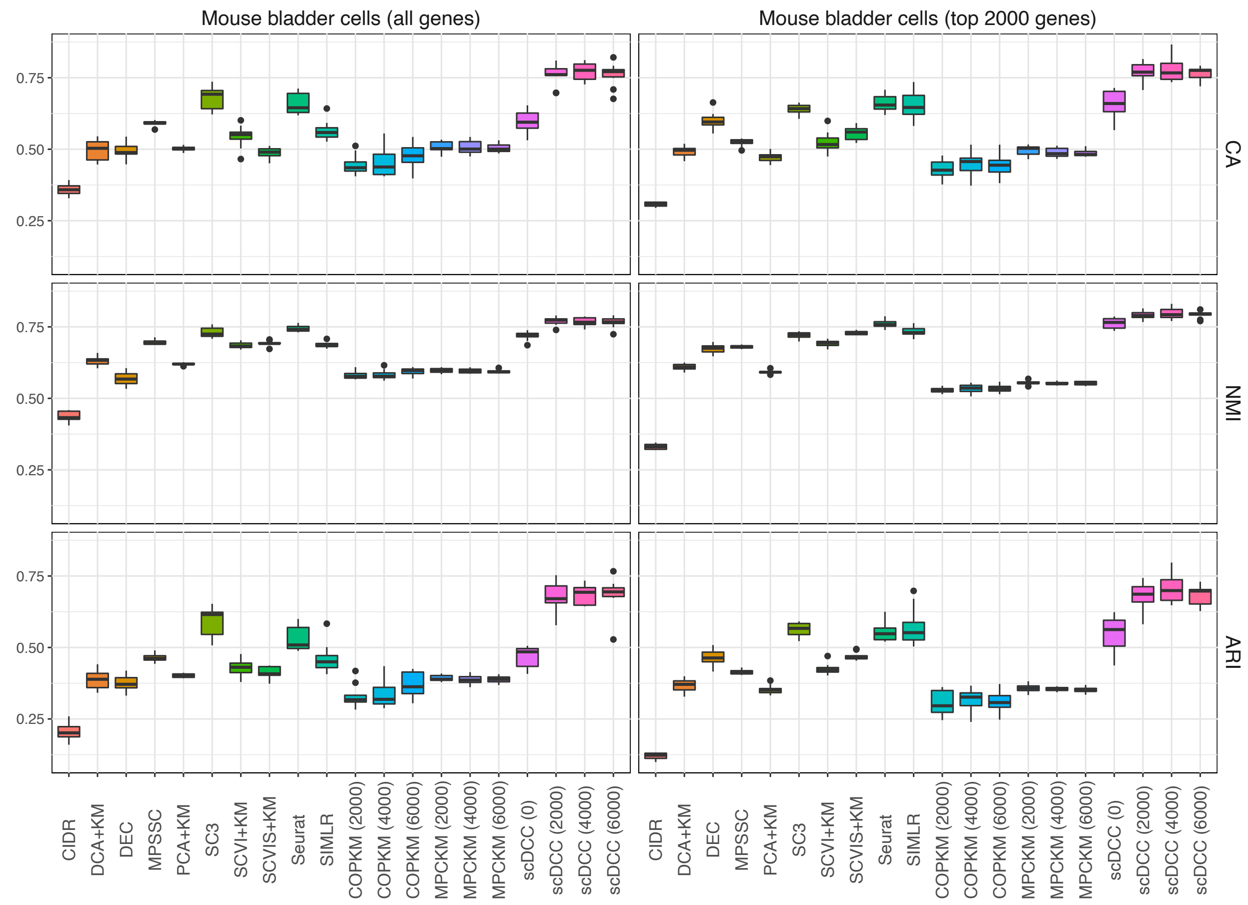


**Figure S3.** Clustering performances of different methods on the Mouse bladder cells (randomly selected 2100 cells), measured by NMI, CA and ARI. The number in parentheses is the number of pairwise constraints. All experiments are repeated for ten times. The lower, middle, and upper hinges correspond to the first, second and third quartiles (the 25th, 50th and 75th percentiles). Let IQR be the distance between the first and third quartiles. The upper whisker extends from the hinge to the largest value no further than 1.5 * IQR from the hinge, while the lower whisker extends from the hinge to the smallest value at most 1.5 * IQR from the hinge. Data points beyond the end of the whiskers are outliers and plotted individually. Note that we use “KM” to represent “k-means” to save space.

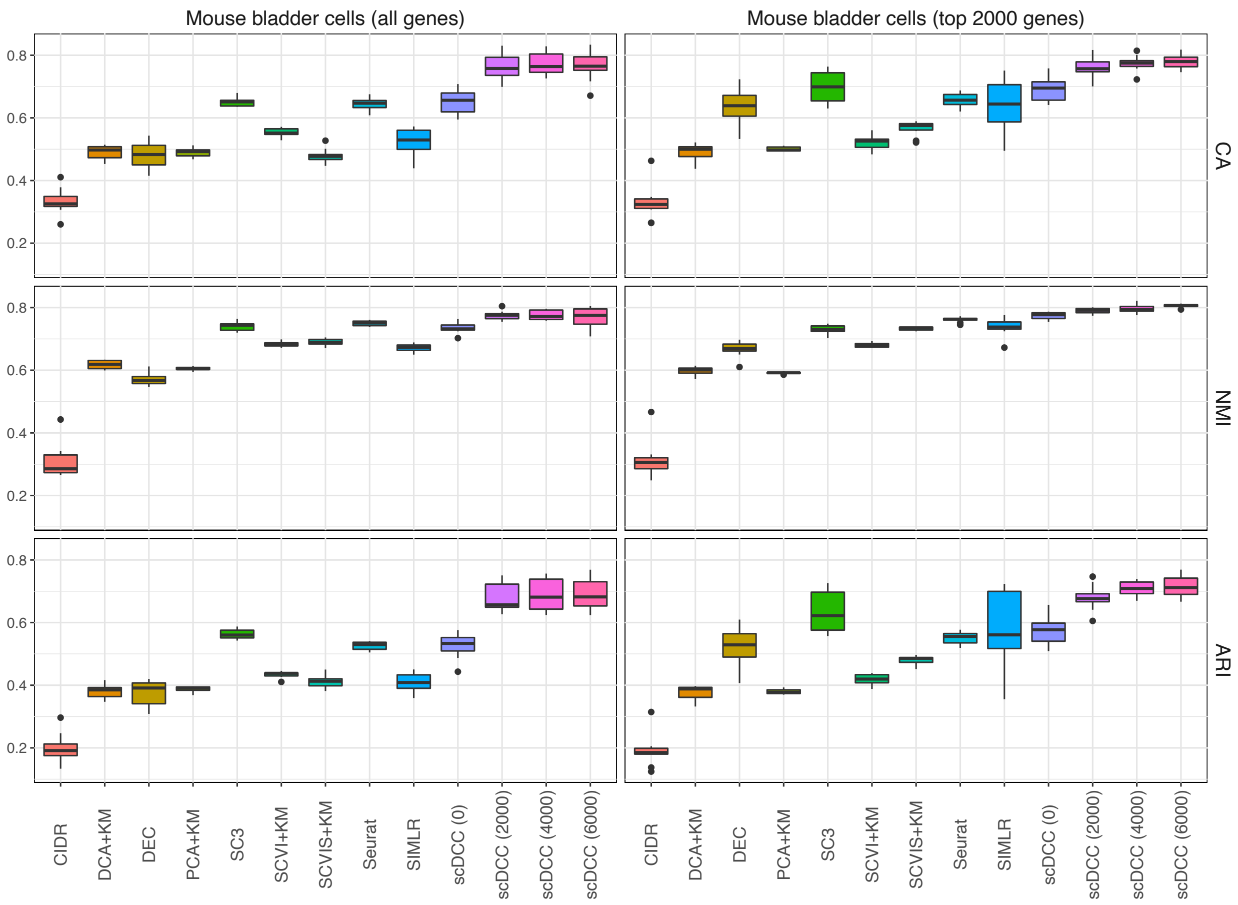


**Figure S4.** Clustering performances of different methods on the Mouse bladder cells (full dataset), measured by NMI, CA and ARI. The number in parentheses is the number of pairwise constraints. All experiments are repeated for ten times. The lower, middle, and upper hinges correspond to the first, second and third quartiles (the 25th, 50th and 75th percentiles). Let IQR be the distance between the first and third quartiles. The upper whisker extends from the hinge to the largest value no further than 1.5 * IQR from the hinge, while the lower whisker extends from the hinge to the smallest value at most 1.5 * IQR from the hinge. Data points beyond the end of the whiskers are outliers and plotted individually. Note that we use “KM” to represent “k-means” to save space.

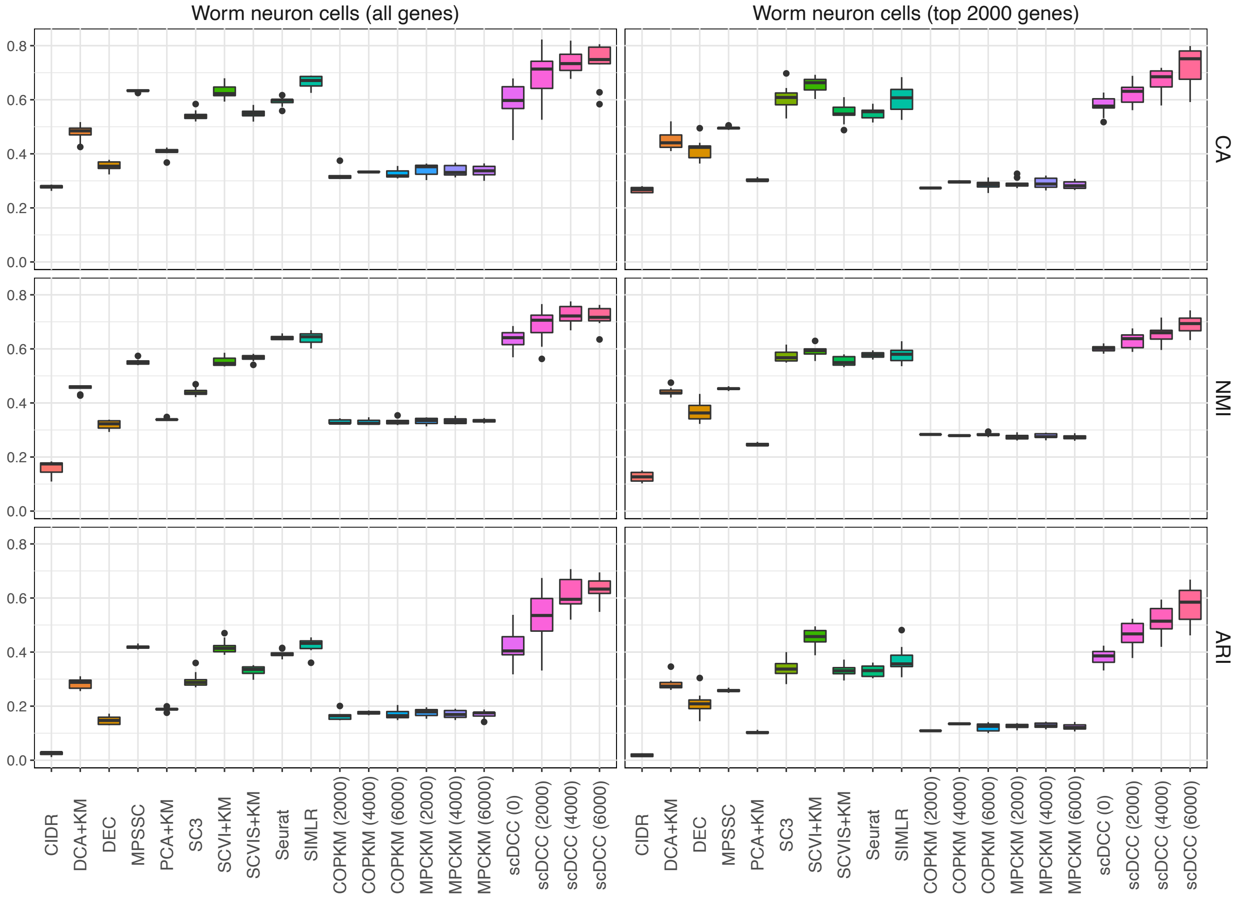


**Figure S5.** Clustering performances of different methods on the Worm neuron cells (randomly selected 2100 cells), measured by NMI, CA and ARI. The number in parentheses is the number of pairwise constraints. All experiments are repeated for ten times. The lower, middle, and upper hinges correspond to the first, second and third quartiles (the 25th, 50th and 75th percentiles). Let IQR be the distance between the first and third quartiles. The upper whisker extends from the hinge to the largest value no further than 1.5 * IQR from the hinge, while the lower whisker extends from the hinge to the smallest value at most 1.5 * IQR from the hinge. Data points beyond the end of the whiskers are outliers and plotted individually. Note that we use “KM” to represent “k-means” to save space.

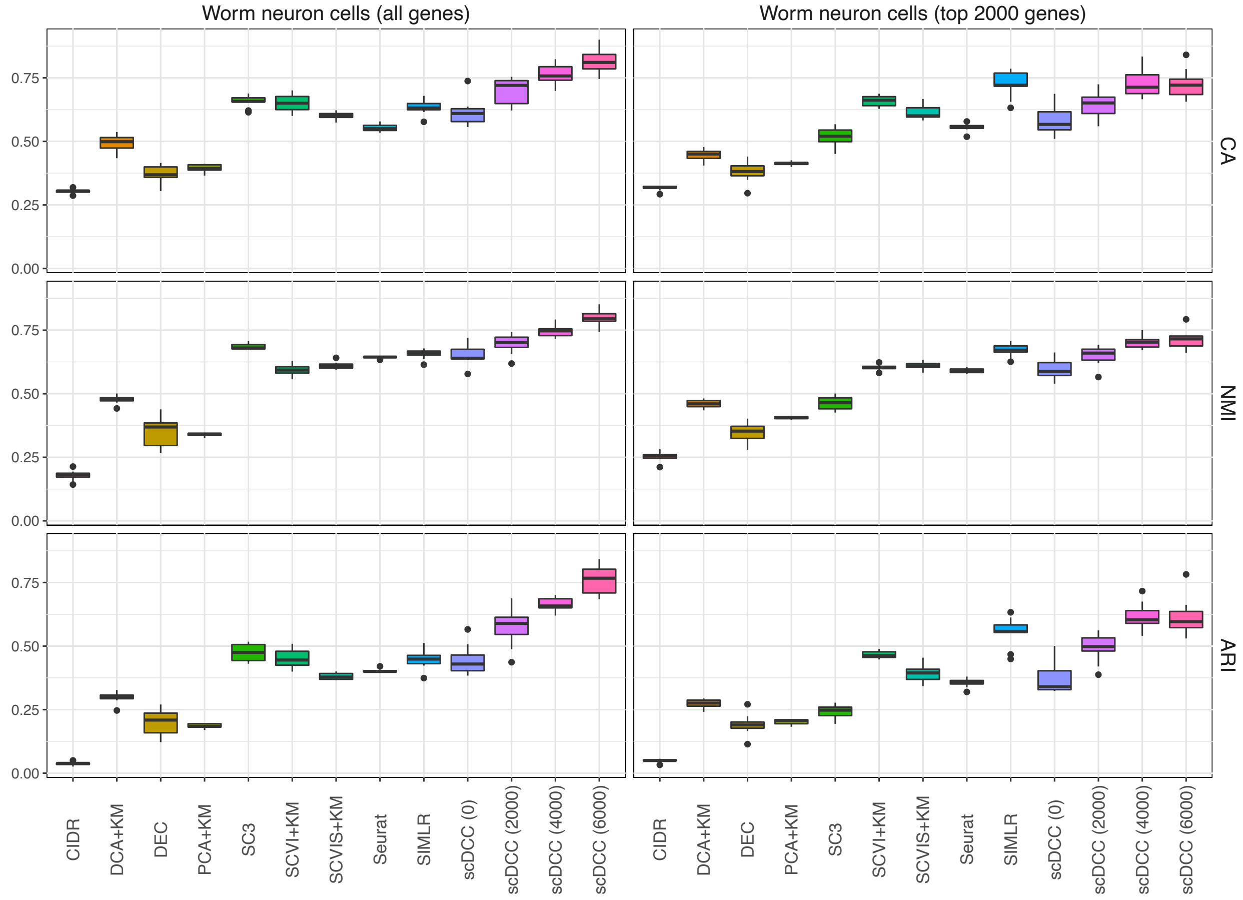


**Figure S6.** Clustering performances of different methods on the Worm neuron cells (full dataset), measured by NMI, CA and ARI. The number in parentheses is the number of pairwise constraints. All experiments are repeated for ten times. The lower, middle, and upper hinges correspond to the first, second and third quartiles (the 25th, 50th and 75th percentiles). Let IQR be the distance between the first and third quartiles. The upper whisker extends from the hinge to the largest value no further than 1.5 * IQR from the hinge, while the lower whisker extends from the hinge to the smallest value at most 1.5 * IQR from the hinge. Data points beyond the end of the whiskers are outliers and plotted individually. Note that we use “KM” to represent “k-means” to save space.

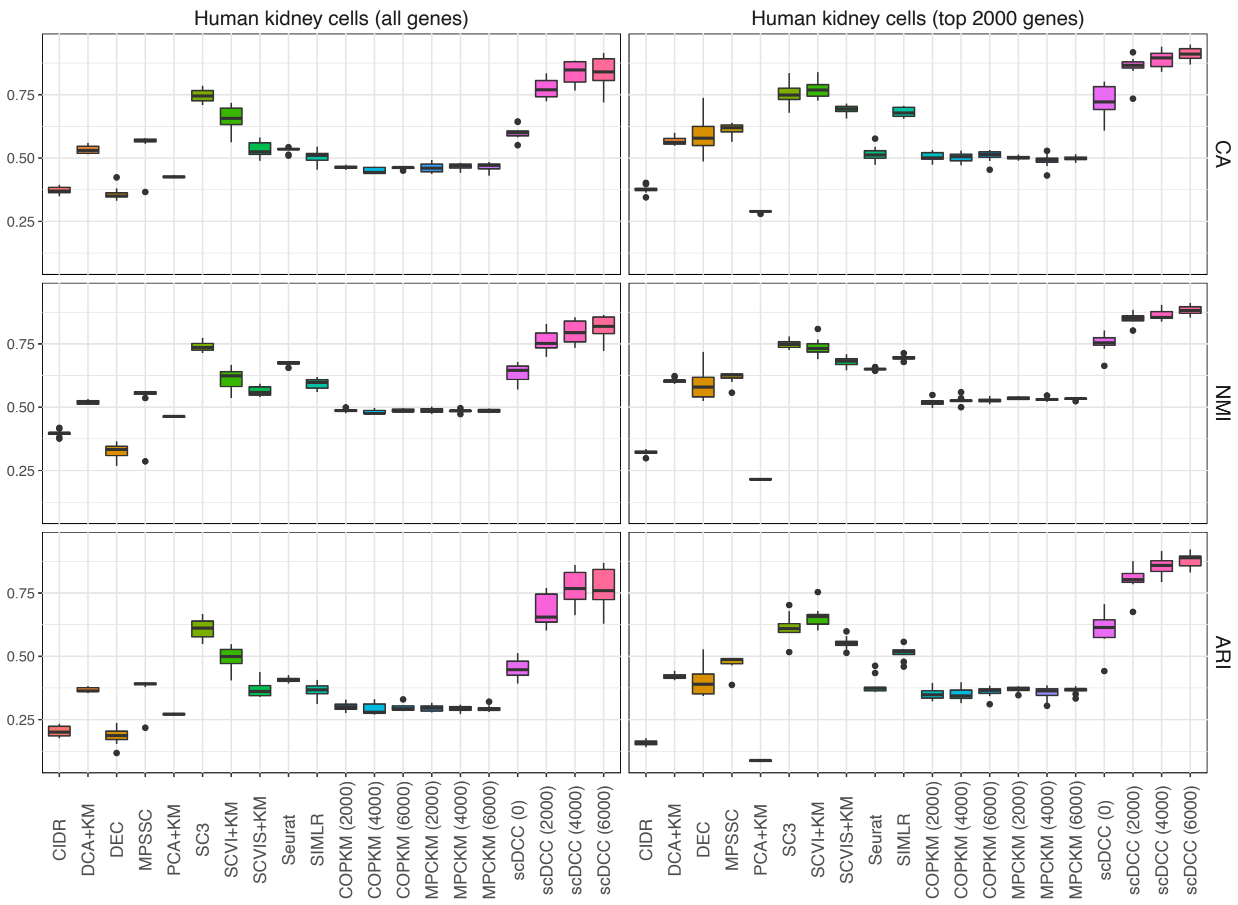


**Figure S7.** Clustering performances of different methods on the Human kidney cells (randomly selected 2100 cells), measured by NMI, CA and ARI. The number in parentheses is the number of pairwise constraints. All experiments are repeated for ten times. The lower, middle, and upper hinges correspond to the first, second and third quartiles (the 25th, 50th and 75th percentiles). Let IQR be the distance between the first and third quartiles. The upper whisker extends from the hinge to the largest value no further than 1.5 * IQR from the hinge, while the lower whisker extends from the hinge to the smallest value at most 1.5 * IQR from the hinge. Data points beyond the end of the whiskers are outliers and plotted individually. Note that we use “KM” to represent “k-means” to save space.

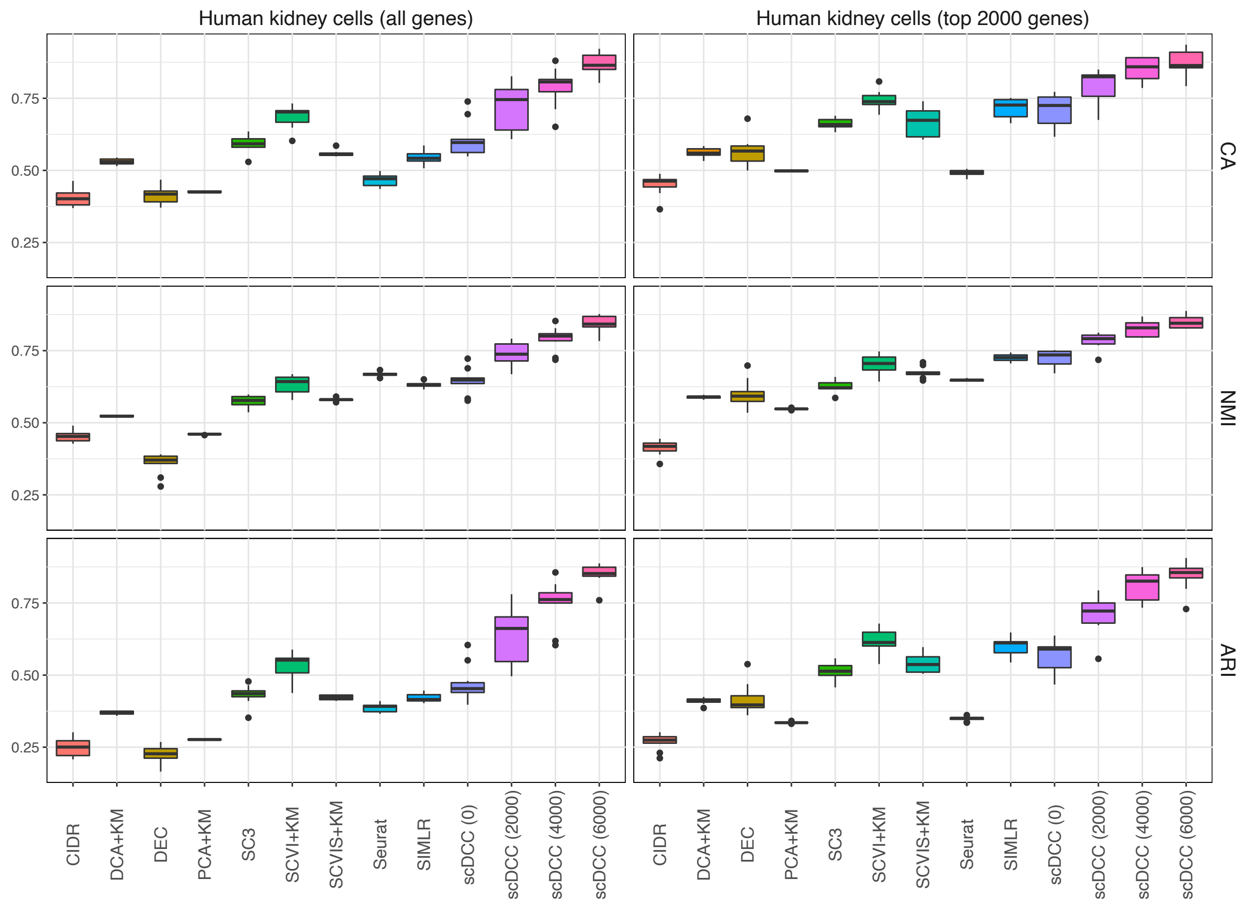


**Figure S8.** Clustering performances of different methods on the Human kidney cells (full dataset), measured by NMI, CA and ARI. The number in parentheses is the number of pairwise constraints. All experiments are repeated for ten times. The lower, middle, and upper hinges correspond to the first, second and third quartiles (the 25th, 50th and 75th percentiles). Let IQR be the distance between the first and third quartiles. The upper whisker extends from the hinge to the largest value no further than 1.5 * IQR from the hinge, while the lower whisker extends from the hinge to the smallest value at most 1.5 * IQR from the hinge. Data points beyond the end of the whiskers are outliers and plotted individually. Note that we use “KM” to represent “k-means” to save space.

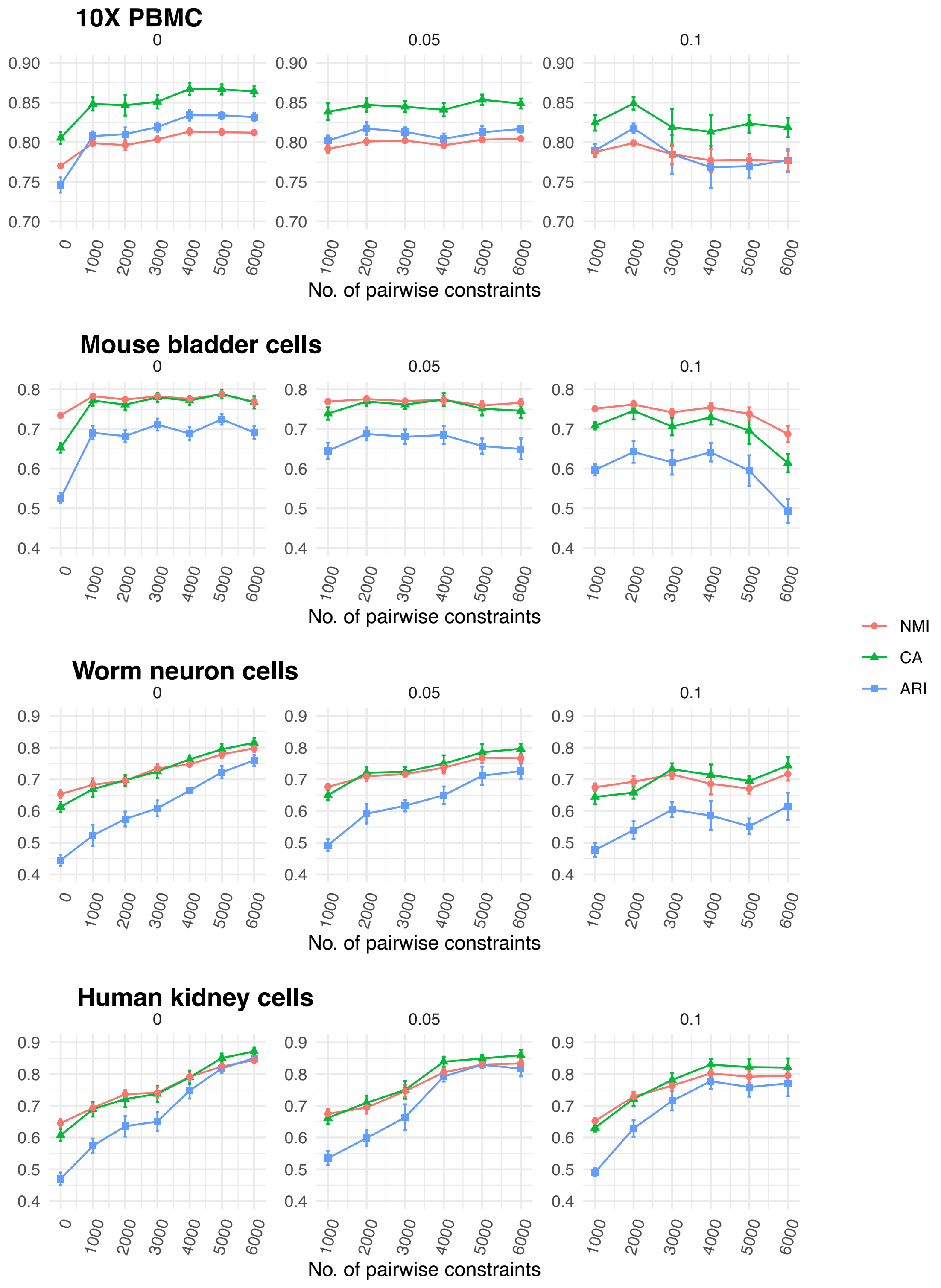


**Figure S9.** Clustering performances of scDCC on four small scRNA-seq datasets (full dataset) with different number of pairwise constraints (left) and noisy constraints (middle, error rate is 5%; right, error rate is 10%), measured by NMI, CA and ARI. All experiments are repeated for ten times, and the mean and standard errors are displayed.

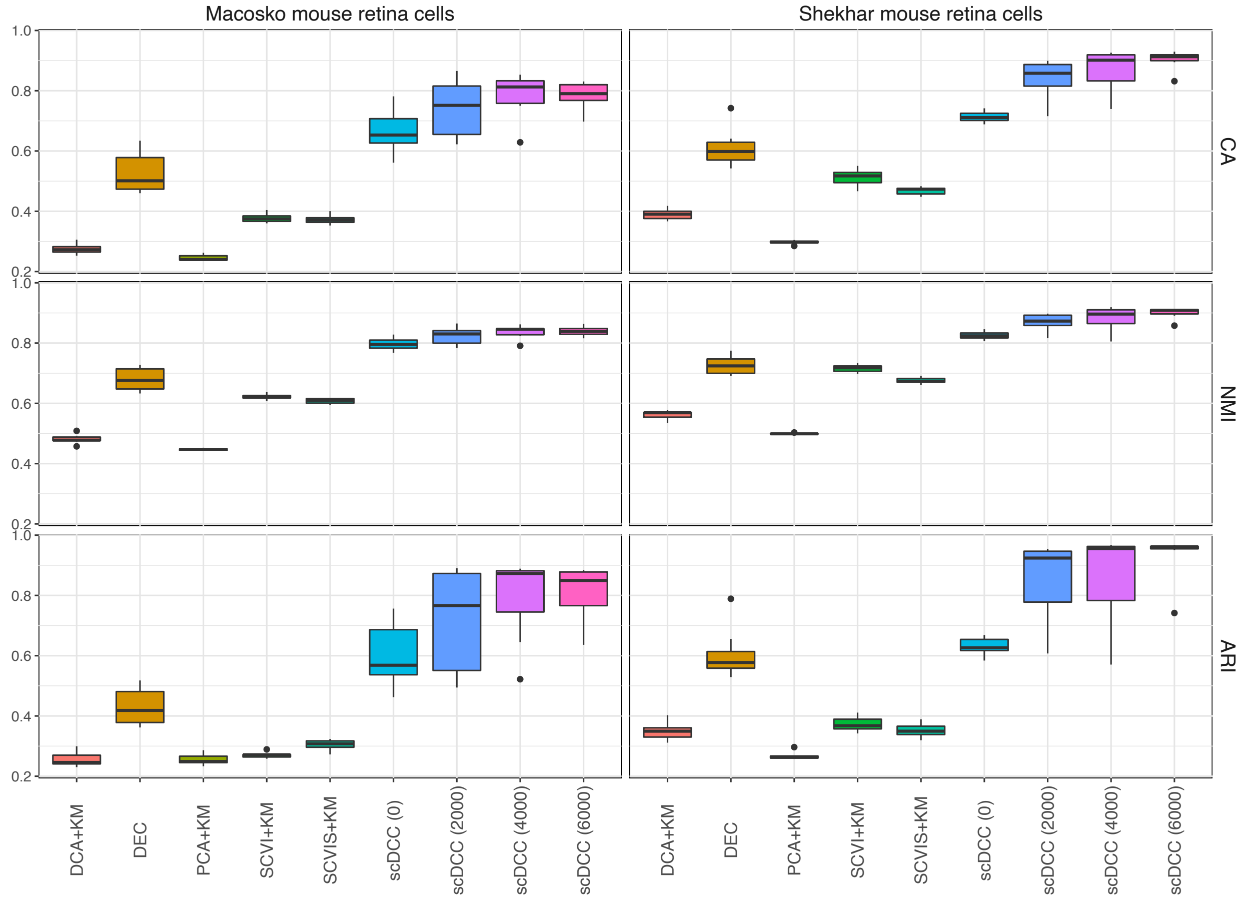


**Figure S10.** Clustering performances of different methods on two large datasets (full dataset), measured by NMI, CA and ARI. The number in parentheses is the number of pairwise constraints. All experiments are repeated for ten times. The lower, middle, and upper hinges correspond to the first, second and third quartiles (the 25th, 50th and 75th percentiles). Let IQR be the distance between the first and third quartiles. The upper whisker extends from the hinge to the largest value no further than 1.5 * IQR from the hinge, while the lower whisker extends from the hinge to the smallest value at most 1.5 * IQR from the hinge. Data points beyond the end of the whiskers are outliers and plotted individually. Note that we use "KM" to represent "k-means" to save space.

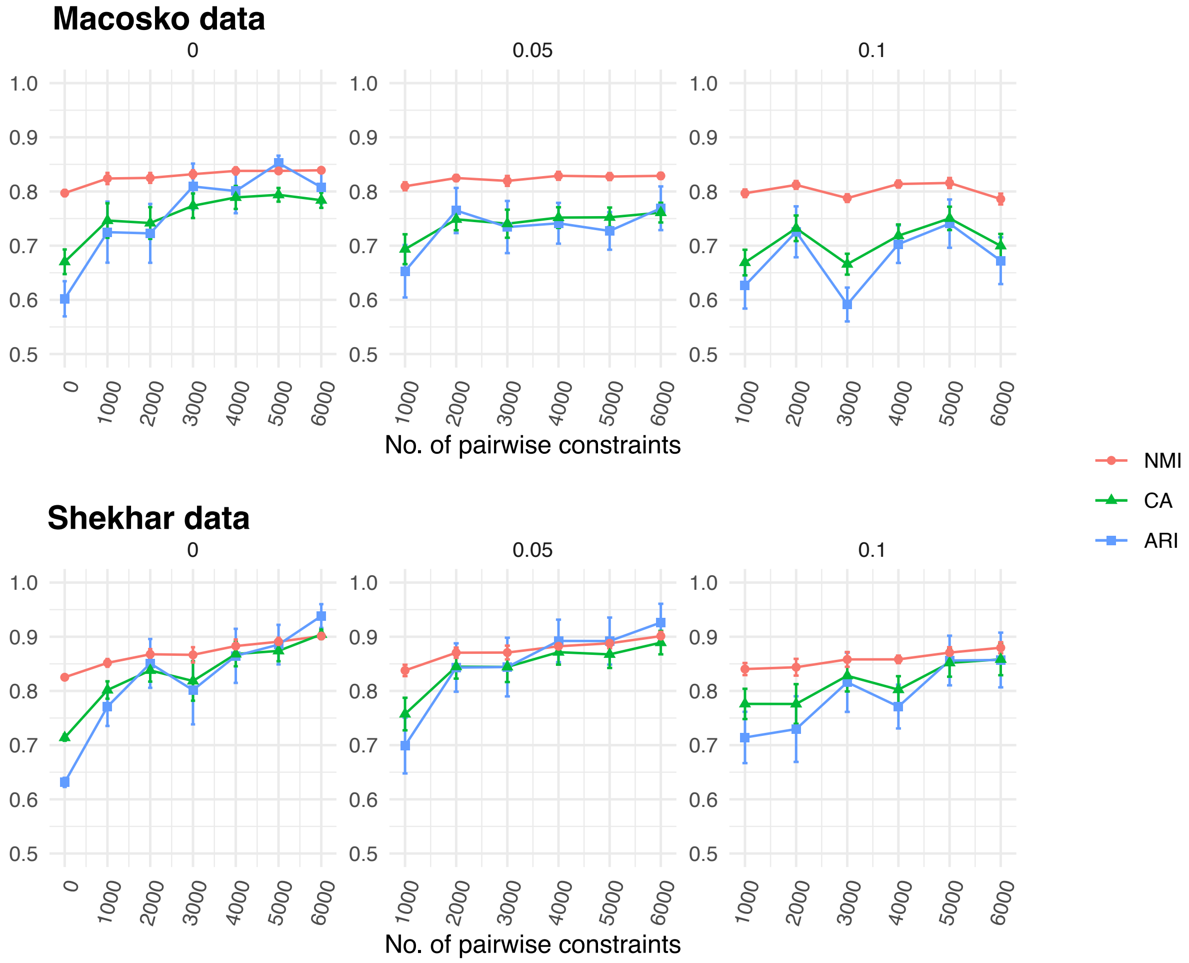


**Figure S11.** Clustering performances of scDCC on two large scRNA-seq datasets with different number of pairwise constraints (left) and noisy constraints (middle, error rate is 5%; right, error rate is 10%), measured by NMI, CA and ARI. All experiments are repeated for ten times, and the mean and standard errors are displayed.

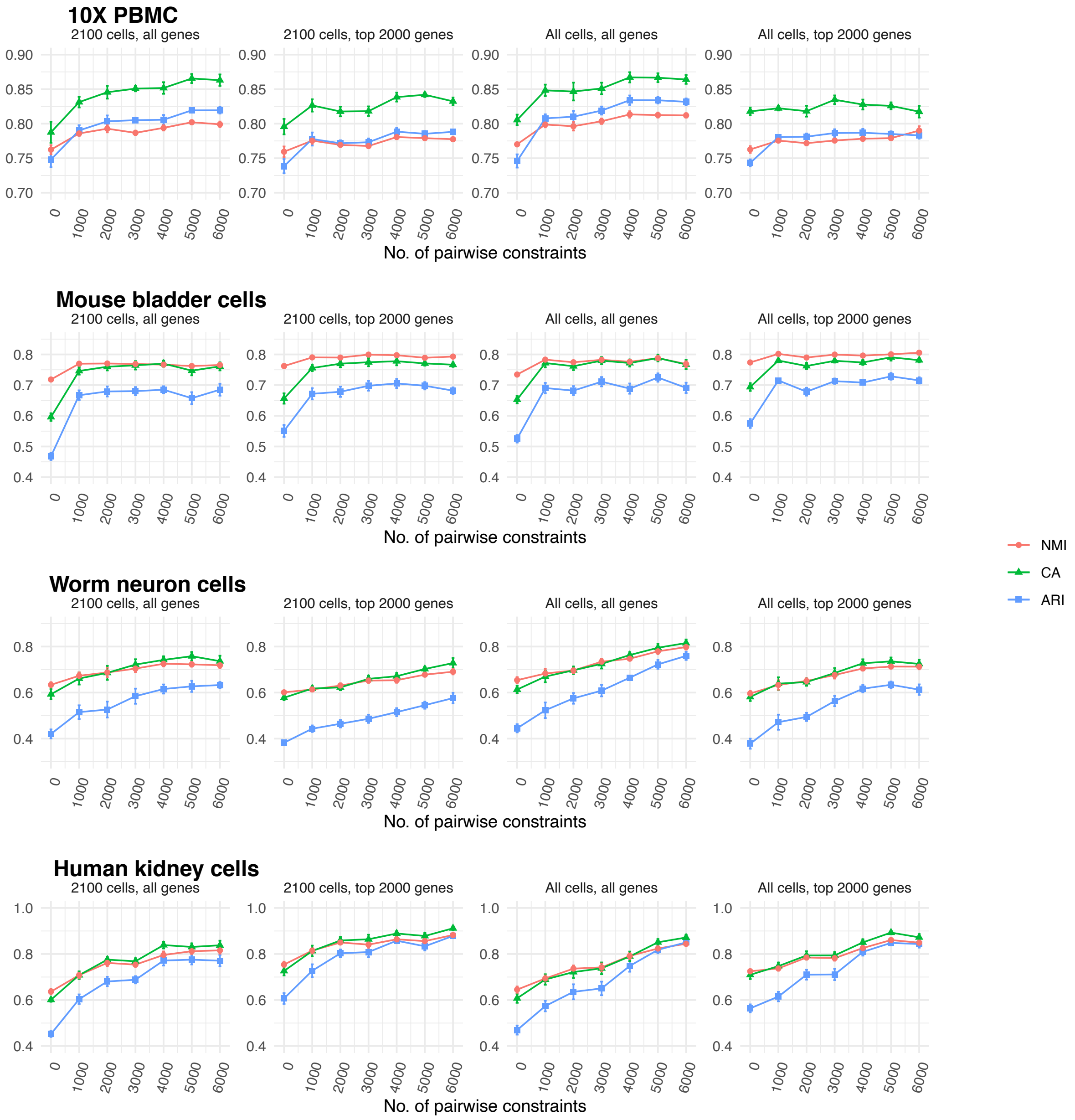


**Figure S12.** Clustering performances of scDCC on four small scRNA-seq datasets with different number of pairwise constraints, measured by NMI, CA and ARI. All experiments are repeated for ten times, and the mean and standard errors are displayed.

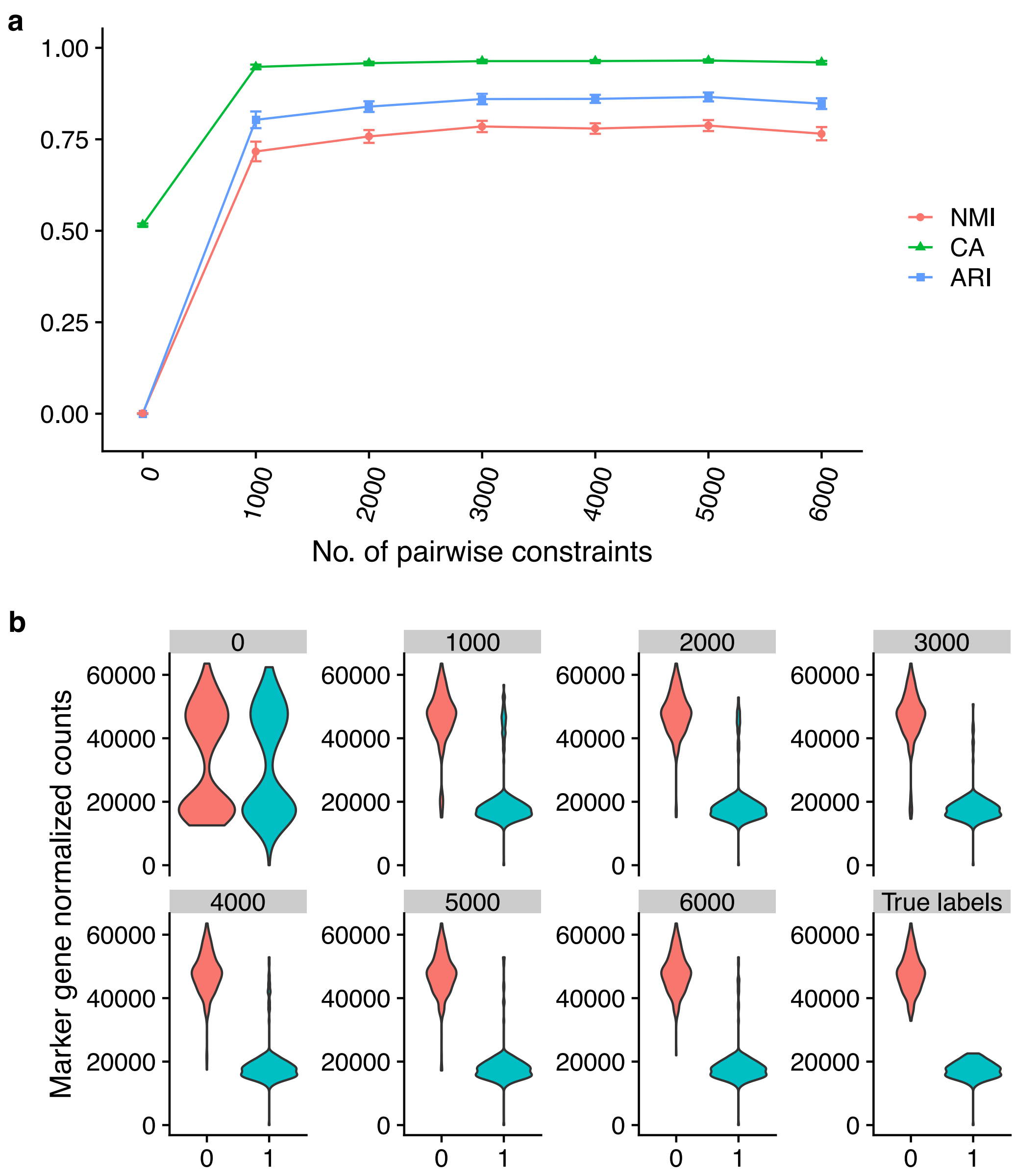


**Figure S13.** Constraints could guide scDCC to separate the marker gene among clusters on simulated data. The data is simulated by Splatter and contains only one marker gene (one gene is differentially expressed among two clusters). The constraints are generated by the marker gene (See details in **Supplementary notes**). **(a)** Clustering performances of scDCC on simulated data with different numbers of marker gene-based pairwise constraints, measured by NMI, CA and ARI. All experiments are repeated for ten times, and the means and standard errors are displayed. **(b)** Violin plots of the marker gene based on labels predicted by scDCC with different number of pairwise constraints (one clustering result of each setting was selected) and true labels. The x axes are clusters and the y axes are normalized counts of the marker gene.

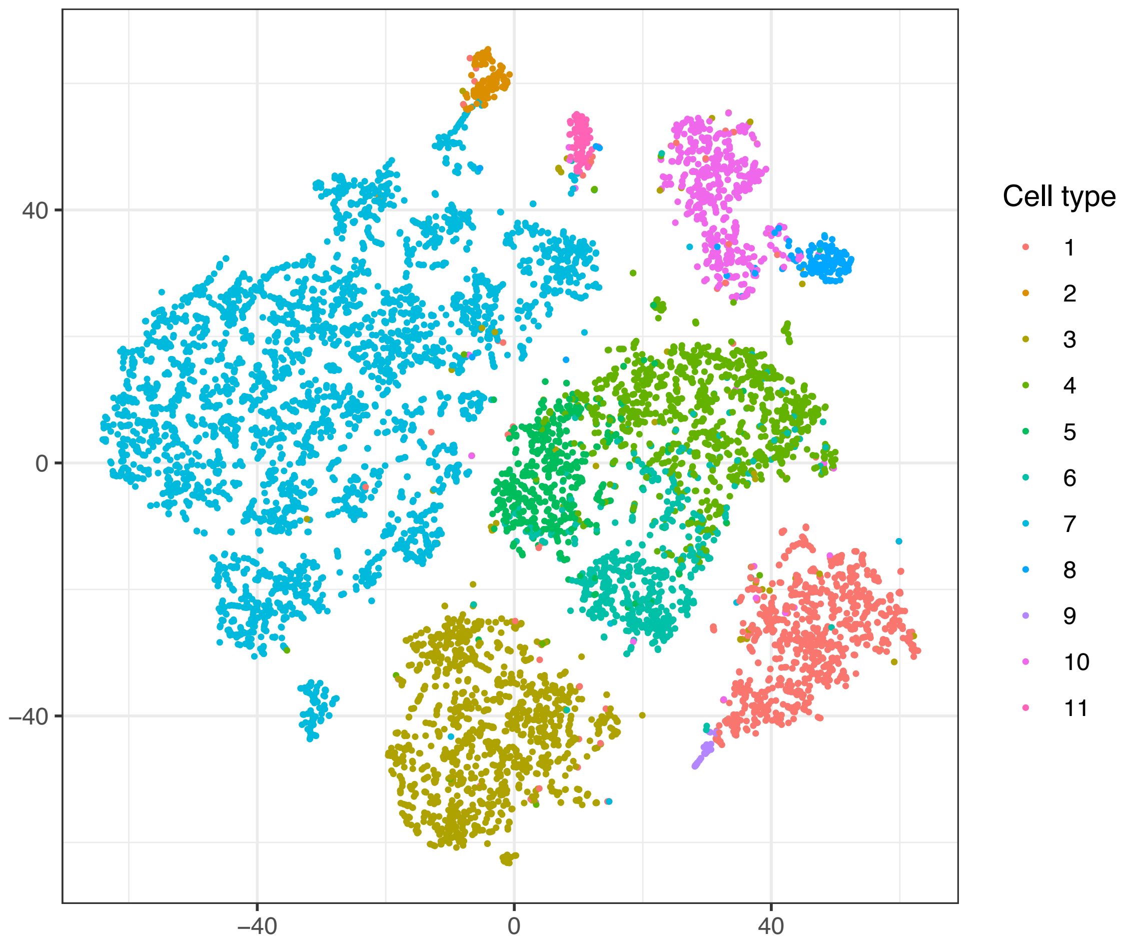


**Figure S14.** t-SNE plot of ZIFA latent features of marker genes in Human liver dataset.

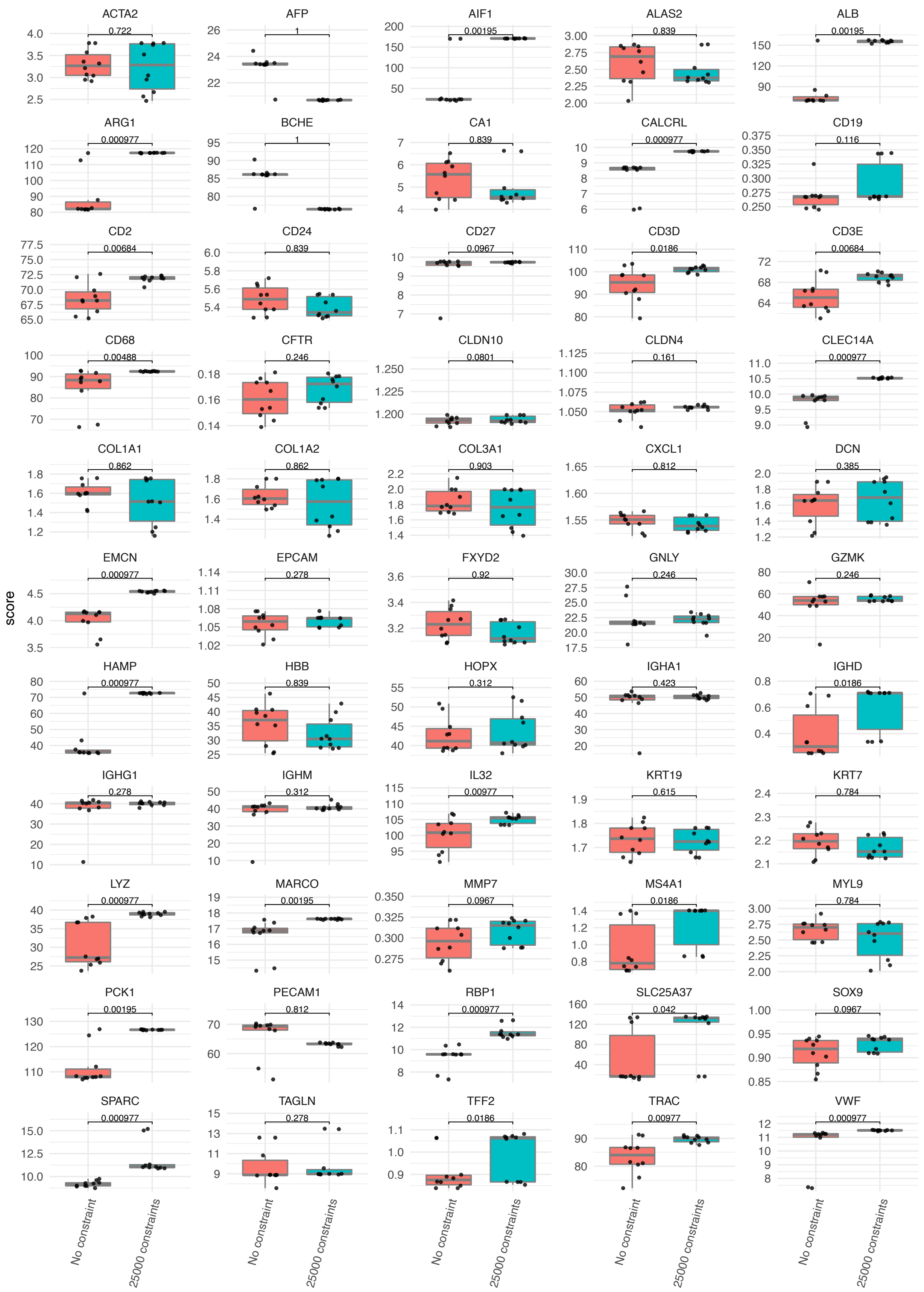
ACTA2
0.722
AFP
1
AIF1
0.00195
ALAS2
0.839
ALB
0.00195
ARG1
0.000977
BCHE
1
CA1
0.839
CALCRL
0.000977
CD19
0.116
CD2
0.00684
CD24
0.839
CD27
0.0967
CD3D
0.0186
CD3E
0.00684
CD68
0.00488
CFTR
0.246
CLDN10
0.0801
CLDN4
0.161
CLEC14A
0.000977
COL1A1
0.862
COL1A2
0.862
COL3A1
0.903
CXCL1
0.812
DCN
0.385
EMCN
0.000977
EPCAM
0.278
FXYD2
0.92
GNLY
0.246
GZMK
0.246
HAMP
0.000977
HBB
0.839
HOPX
0.312
IGHA1
0.423
IGHD
0.0186
IGHG1
0.278
IGHM
0.312
IL32
0.00977
KRT19
0.615
KRT7
0.784
LYZ
0.000977
MARCO
0.00195
MMP7
0.0967
MS4A1
0.0186
MYL9
0.784
PCK1
0.00195
PECAM1
0.812
RBP1
0.000977
SLC25A37
0.042
SOX9
0.0967
SPARC
0.000977
TAGLN
0.278
TFF2
0.0186
TRAC
0.00977
VWF
0.000977
score
No constraint
25000 constraints

**Figure S15.** Cluster specificity scores of the 55 marker genes in Human liver dataset with different constraints. Ten repeats are displayed. All plots are standard boxplots, which display the distribution of data by presenting the inner fence (the whisker, taken to 1.5× the Inter Quartile range, or IQR, from the quartile), first quartile, median, third quartile and outliers. Differences of specificity scores between without and with constraints are quantified by one-sided Wilcoxon p-values.

# Supplementary Notes

## Simulation experiment

The simulated data is generated by the Splatter R package [1]. We generated 500 cells by 100 genes, two clusters, with the setting of *dropout.mid* = 1, *de.facScale* = 0.5, *de.prob* = 0.01 and *de.downProb* = 0. As a result, there was only one gene that was expressed differently among the two clusters, and this gene was defined as the marker gene. After generating the data, we obtained “pseudo” labels based on normalized counts of the marker gene (normalized by the library size). If normalized counts of the marker gene > 75 quantile, then we defined the “pseudo” label as 0; if normalized counts of the marker gene < 25 quantile, then we defined the “pseudo” label as 1. Pairwise constraints were generated by “pseudo” labels. Since the data only had 100 genes, we modified the scDCC model such that the encoder and the decoder had one hidden layer of size 32, and the bottleneck layer had the size of 2. We randomly generated different numbers of pairwise constraints and repeated experiments on each setting for ten times.

## References

1. Zappia L, Phipson B, Oshlack A. Splatter: simulation of single-cell RNA sequencing data. *Genome Biol* **18**, 174 (2017).
